# Review of cobalamin status and disorders of cobalamin metabolism in dogs

**DOI:** 10.1111/jvim.15638

**Published:** 2019-11-23

**Authors:** Stefanie Kather, Niels Grützner, Peter H. Kook, Franziska Dengler, Romy M. Heilmann

**Affiliations:** ^1^ Department for Small Animals, Veterinary Teaching Hospital, College of Veterinary Medicine University of Leipzig Leipzig Germany; ^2^ Institute of Agricultural and Nutritional Sciences Martin Luther University Halle‐Wittenberg Halle (Saale) Germany; ^3^ School of Veterinary Science Massey University Palmerston North New Zealand; ^4^ Clinic for Small Animal Internal Medicine, Vetsuisse Faculty University of Zurich Zurich Switzerland; ^5^ Institute of Veterinary Physiology, College of Veterinary Medicine University of Leipzig Leipzig Germany

**Keywords:** cobalamin deficiency, cubam receptor, folate, homocysteine, hypercobalaminemia, hypocobalaminemia, methylmalonic acid, vitamin B_12_

## Abstract

Disorders of cobalamin (vitamin B_12_) metabolism are increasingly recognized in small animal medicine and have a variety of causes ranging from chronic gastrointestinal disease to hereditary defects in cobalamin metabolism. Measurement of serum cobalamin concentration, often in combination with serum folate concentration, is routinely performed as a diagnostic test in clinical practice. While the detection of hypocobalaminemia has therapeutic implications, interpretation of cobalamin status in dogs can be challenging. The aim of this review is to define hypocobalaminemia and cobalamin deficiency, normocobalaminemia, and hypercobalaminemia in dogs, describe known cobalamin deficiency states, breed predispositions in dogs, discuss the different biomarkers of importance for evaluating cobalamin status in dogs, and discuss the management of dogs with hypocobalaminemia.

**Abbreviations**
AMN: amnionless
CIE: chronic inflammatory enteropathies
CUBN: cubilin
EPI: exocrine pancreatic insufficiency
GC/MS: gas chromatography‐mass spectrometry
GI: gastrointestinal
HCY: homocysteine
IBD: inflammatory bowel disease
IF: intrinsic factor
IGS: Imerslund‐Gräsbeck syndrome
IRE: immunosuppressant‐responsive enteropathy
LC/MS‐MS: liquid chromatography‐tandem mass spectrometry
LoQ: lower limit of quantification
MMA: methylmalonic acid
NRE: nonresponsive enteropathy
PLE: protein‐losing enteropathy
RI: reference interval
SI: small intestine

## 1 INTRODUCTION

Disorders of cobalamin (vitamin B_12_) metabolism are increasingly recognized in small animal medicine. Causes of cobalamin deficiency in dogs vary from chronic gastrointestinal (GI) disease to hereditary defects in cobalamin metabolism. Measurement of serum cobalamin concentration, often in combination with serum folate concentration, is routinely performed as a diagnostic test in small animal practice to detect dogs with vitamin B deficiency. The detection of hypocobalaminemia also has therapeutic implications. Because interpretation of the cobalamin status in dogs and the management of dogs with a suboptimal cobalamin status can be challenging, an update on this topic and a definition of the different cobalamin states in dogs are provided here.

The aim of this review is to critically evaluate the veterinary literature to define serum cobalamin status (normocobalaminemia, hypocobalaminemia and cobalamin deficiency, and hypercobalaminemia), describe causes of cobalamin deficiency states in dogs including breed predispositions, summarize breed‐specific changes in cobalamin metabolism, discuss biomarkers of importance for evaluating cobalamin status in dogs, and discuss the treatment options for dogs in which a suboptimal or deficient cobalamin status has been diagnosed.

## 2 COBALAMIN METABOLISM IN DOGS

### 2.1 Ingestion and absorption of cobalamin

Cobalamin is a water‐soluble vitamin, also referred to as vitamin B_12_. It is mainly ingested with food of animal origin with liver, kidney, meat, egg, milk products as well as fish having high cobalamin content, but smaller amounts can also be produced by the intestinal microbiota.^1^

**Figure 1:**
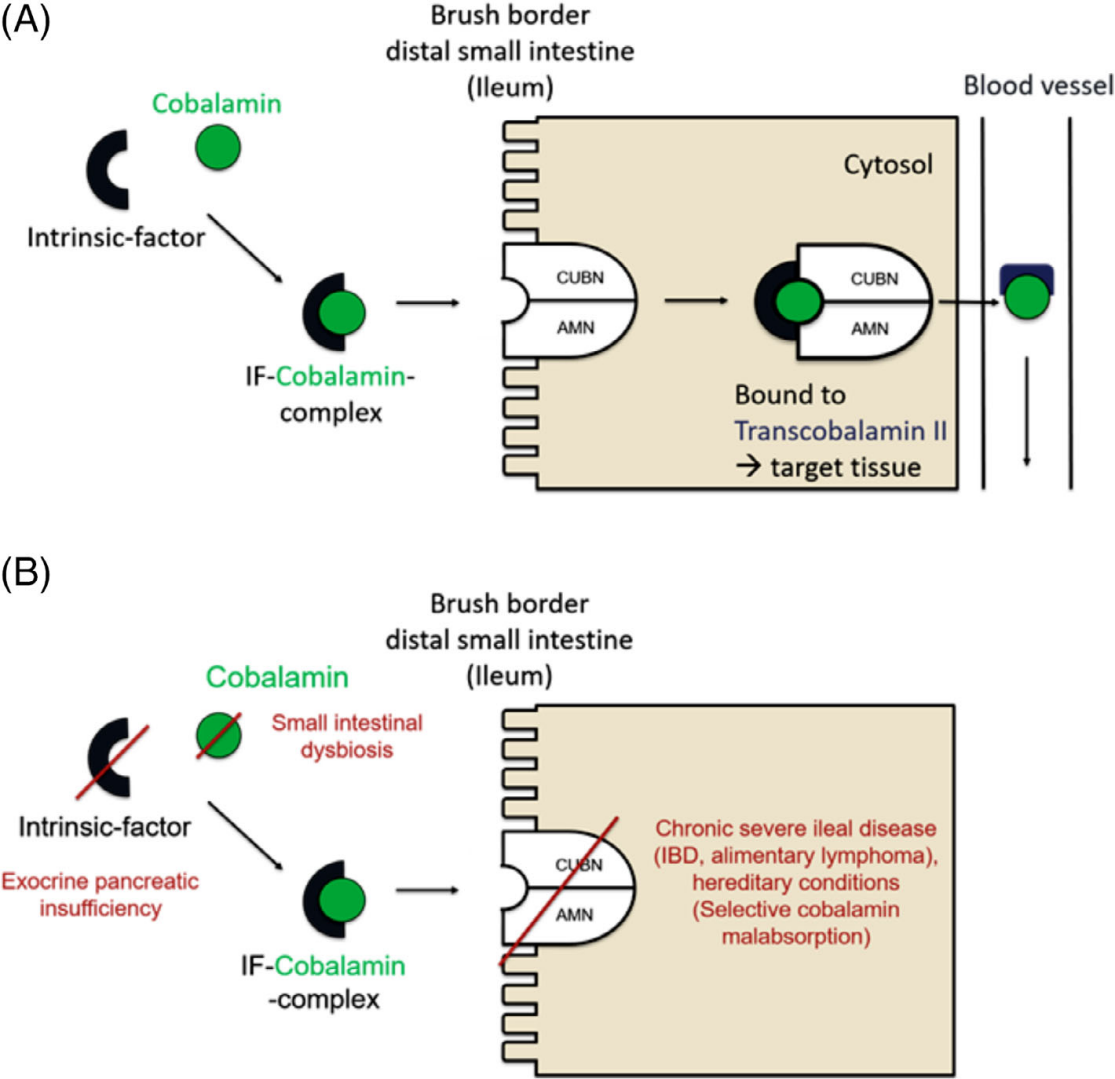
Schematic of the absorption of cobalamin by enterocytes in the distal small intestine (ileum). A, In the duodenum cobalamin is bound to intrinsic factor (IF). The cobalamin‐IF complex is then absorbed by receptor‐mediated endocytosis. This cubam receptor is localized at the brush border of the distal small intestine (ileum). The receptor complex is comprised of 2 subunits, the proteins amnionless (AMN) and cubilin (CUBN). Within the lysosomes of the enterocytes, cobalamin is separated from IF and the receptor. Bound to the transport protein transcobalamin II, cobalamin is then transported within the bloodstream to its target tissues. A small amount of circulating cobalamin is bound to transcobalamin I and thus unavailable for cellular uptake. Approximately 1% of dietary cobalamin is absorbed via passive diffusion across the intestinal mucosa. B, Various disorders can impair cobalamin metabolism

**Figure 2:**
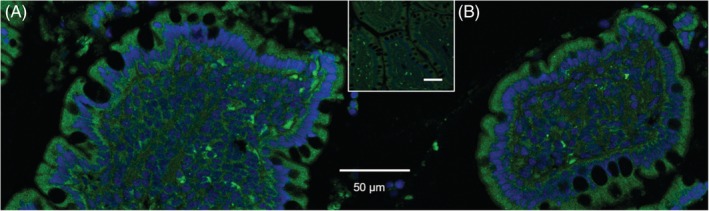
Immunofluorescent staining of the cobalamin receptor subunits in the canine ileum epithelium. The expression of the cobalamin receptor was detected immunohistochemically in a cross section of an ileal villus from a dog using antibodies for the subunits (A) amnionless (AMN, green) and (B) cubilin (CUBN, green). Both subunits are localized throughout the entire enterocyte (cell membrane and cytosol). Nuclei are stained in blue with diamidine phenylindole (DAPI). The insert shows the corresponding secondary antibody control. Images were acquired using equipment at the Laser Scanning Microscopy Core Facility (College of Veterinary Medicine, University of Leipzig, Germany)

**Figure 3:**
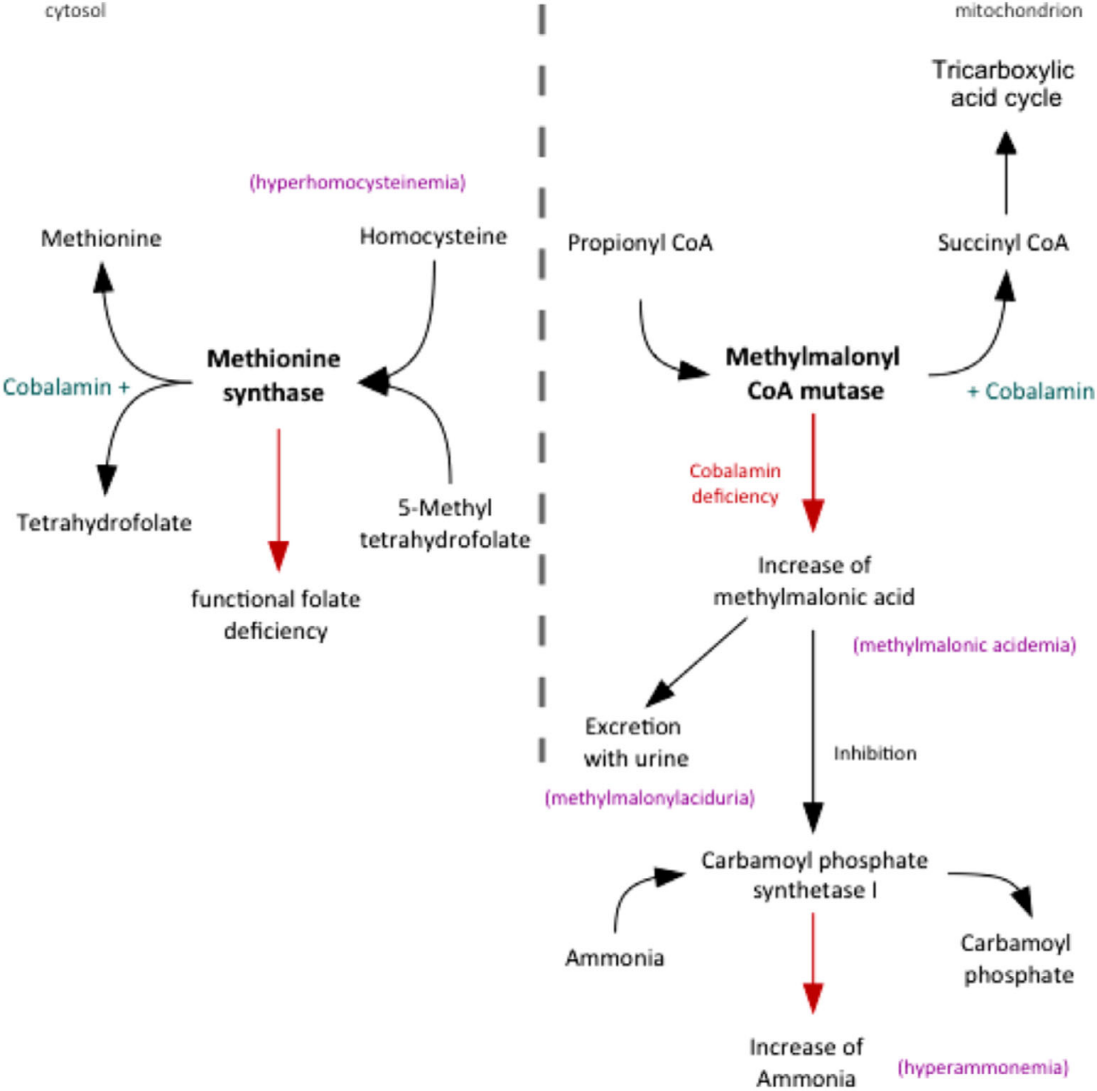
Intracellular pathways of cobalamin metabolism. *Methionine synthase* catalyzes the regeneration of methionine from homocysteine. Disorders associated with a deficiency in cellular cobalamin availability thus can lead to functional folate deficiency and increased concentrations of homocysteine. *Methylmalonic CoA mutase* catalyzes the reaction from methylmalonyl CoA to succinyl CoA which is a key molecule in the tricarboxylic acid cycle. A lack of intracellular cobalamin leads to a reduced enzyme activity and an accumulation of methylmalonic acid (MMA). Excess MMA is excreted in the urine. MMA can also inhibit the activity of carbamoyl phosphate synthetase I, an enzyme of the urea cycle. Carbamoyl phosphate synthetase I normally metabolizes ammonia to carbamoyl phosphate. When this metabolic process is impaired, plasma ammonia concentration increases

Plants and plant products contain virtually no cobalamin and, whereas ruminants and other herbivores can produce a sufficient amount of cobalamin in their intestines, omnivorous or carnivorous animals including dogs and cats are not able to produce cobalamin. The intestinal microbiota of dogs and cats can produce cobalamin in the presence of cobalt, but because this site of cobalamin production is distal to the site of its absorption, this ability appears to be of little benefit to the animal. Most commercial pet foods, including vegan or vegetarian diets, are supplemented with cobalamin,^2^, ^3^ but the content of dietary cobalamin varies between diets (dry diets: 0.05‐0.25 mg/kg dry matter basis; canned diets: 0.03‐0.59 mg/kg dry matter basis).^4^ The National Research Council (NRC 2006) recommendation for cobalamin fortification is 35 μg/kg dry matter for canine diets, regardless of the canine life stage.^5^ The concentration of serum cobalamin and cobalamin‐dependent metabolites is not related to the dogs being fed a biologically appropriate raw food diet or a commercial dog food.^6^ Bound to dietary protein, cobalamin reaches the stomach where it is released by activated pepsinogen and gastric acid.^7^ Free cobalamin is then bound to haptocorrin (R protein, transcobalamin I) to protect it from bacterial utilization in the proximal GI tract.^7^ In the duodenum, pancreatic proteases separate cobalamin from haptocorrin, and free cobalamin is bound to intrinsic factor (IF). The major site of IF synthesis is the gastric mucosa in humans, whereas in dogs IF is produced primarily by the exocrine pancreas and to a lesser extent in the stomach.^8^, ^9^ The cobalamin‐IF‐complex is then absorbed by receptor‐mediated endocytosis. The receptor, known as cubam, is localized at the brush border of the ileum (Fig. 1A and 2). This receptor complex is comprised of 2 subunits, the proteins amnionless (AMN) and cubilin (CUBN) (Fig. 1A and 2).^10^ In addition to receptor‐mediated cobalamin uptake at the ileal brush border epithelium, approximately 1% of dietary cobalamin is absorbed via passive diffusion across the intestinal mucosal epithelium, assumed to occur along the entire length of the GI tract.^11^ Within the lysosomes of the enterocytes, cobalamin is separated from IF and the receptor. Bound to another transport protein, transcobalamin, cobalamin is then transported within the bloodstream to its target tissues. In humans, 20%‐30% of the cobalamin is bound to transcobalamin II, whereas most of the circulating cobalamin (70%‐80%) is bound to transcobalamin I and thus unavailable for cellular uptake.^12^, ^13^ Although transcobalamin II appears to be much more abundant in dogs than in humans, transcobalamin I exists in dogs.^14^ At the target tissue, cobalamin enters the cells through specific receptors and is then released from transcobalamin II (Fig. 1A).^12^ Approximately 15 μg/day of recirculating cobalamin is extracted by hepatocytes and secreted in bile bound to haptocorrin for enterohepatic recirculation.^15^ Within all eukaryotic cells, cobalamin acts as an essential cofactor for the intracellular enzymes methionine synthase and methylmalonyl‐CoA mutase (Fig. 3).^16^

### 2.2 Intracellular function

#### 2.2.1 Methionine synthase

Methionine synthase, a cobalamin‐, folate‐, and pyridoxine (vitamin B_6_)‐dependent enzyme, catalyzes the regeneration of methionine from homocysteine (HCY). This reaction links both cobalamin and folate metabolism (Fig. 3). Disorders associated with a deficiency in cellular cobalamin availability can thus lead to functional folate deficiency states and increased serum concentrations of HCY (hyperhomocysteinemia).

#### 2.2.2 Methylmalonyl‐CoA mutase

Methylmalonyl‐CoA mutase, a cobalamin‐dependent enzyme, catalyzes the formation of succinyl‐CoA from methylmalonyl‐CoA, which is produced by the catabolism of odd‐chain fatty acids and amino acids (Fig. 3). Succinyl‐CoA is a key molecule in the citric acid cycle. A lack of intracellular cobalamin leads to a reduced enzyme activity and an accumulation of methylmalonic acid (MMA) intracellularly and subsequently also systemically (methylmalonic acidemia). On the one hand, excess MMA undergoes urinary excretion (methylmalonic aciduria); on the other hand, MMA can also inhibit the activity of carbamoyl phosphate synthetase I, an enzyme of the urea cycle. Carbamoyl phosphate synthetase I normally metabolizes ammonia to carbamoyl phosphate. When this metabolic process is impaired, plasma ammonia concentrations typically increase.^1^, ^17^, ^18^, ^19^, ^20^ Neurological disorders can thus be a complication of cobalamin deficiency states in dogs due to the increase in systemic MMA concentrations.^1^, ^17^, ^18^, ^21^ In humans, neurological symptoms also occur in organic acidemias including methylmalonic acidemia.^22^

### 2.3 Excretion of cobalamin

Body stores of cobalamin in dogs far exceed the amount of cobalamin that is normally lost through the intestinal tract. Cobalamin undergoes biliary excretion (bound to haptocorrin), and a large amount of cobalamin is conserved by enterohepatic recirculation.^1^

Renal glomerular filtration of the transcobalamin II‐cobalamin complex is followed by tubular reabsorption to minimize urinary losses of cobalamin. Megalin, an endocytic receptor in the proximal renal tubulus that has a high affinity for the transcobalamin II‐cobalamin complex, mediates renal reabsorption and retention of cobalamin. Free cobalamin is excreted in the urine.^23^, ^24^ The cubam receptor is also present in the kidneys^25^ and is involved in the renal reabsorption of several proteins (eg, albumin, transferrin, and vitamin D‐binding protein).

## 3 LABORATORY TESTING TO EVALUATE COBALAMIN STATUS

Subnormal serum cobalamin concentration or cellular cobalamin deficiency in dogs can be detected by measuring serum cobalamin, HCY, and MMA concentrations. Although serum cobalamin concentrations are measured routinely in dogs and cats and determination of serum cobalamin is offered by a number of veterinary diagnostic laboratories, quantification of serum HCY and MMA is not routinely available, although both are considered as useful markers to determine the cellular cobalamin status of companion animals.^7^, ^26^, ^27^, ^28^

### 3.1 Laboratory tests

#### 3.1.1 Serum cobalamin concentration

There are several commercially available immunoassays for the measurement of serum cobalamin concentrations. An automated chemiluminescence assay is routinely used in North America and Europe, and the reference intervals (RI) for serum cobalamin concentrations in dogs that are used by the different veterinary diagnostic laboratories are similar (Table 1). An immunoassay is also available for measurement of serum cobalamin concentrations in dogs but yields different results than the chemiluminescent assay.^29^

**Table 1:** Reference intervals (RIs) for serum cobalamin concentration

	RI for serum cobalamin concentration		Assay working range
	Lower reference limit	Upper reference limit	Lower limit of quantification
Texas A&M University Gastrointestinal Laboratory	251 ng/L (185 pmol/L)	908 ng/L (670 pmol/L)	149 ng/L (110 pmol/L)
Idexx, Germany	317 ng/L (234 pmol/L)	1100 ng/L (812 pmol/L)	45 ng/L (33 pmol/L)
Synlab, Germany	299 ng/L (221 pmol/L)	801 ng/L (591 pmol/L)	150 ng/L (111 pmol/L)
Laboklin, Germany	301 ng/L (222 pmol/L)	802 ng/L (592 pmol/L)	50 ng/L (37 pmol/L)

Note: Shown are the RIs for serum cobalamin concentrations in dogs that have been established by 4 selected international veterinary diagnostic laboratories. The RIs used by these 4 diagnostic laboratories are comparable.

The main indication for measuring serum cobalamin is to identify a subnormal cobalamin status.^30^ Because cobalamin‐dependent metabolic reactions are localized to the intracellular compartment (ie, cytoplasm and mitochondria), the serum cobalamin concentration does not necessarily exactly reflect the whole‐body cobalamin status in an individual dog. Intracellular storage of cobalamin is mostly as a cofactor bound to cobalamin‐dependent enzymes that might or might not be saturated with cobalamin. Decreased liver and kidney cobalamin stores, and certain defects in cobalamin metabolism, such as malabsorption due a defect in the ileal receptor, can result in cobalamin deficiency. Therefore, other markers that more closely reflect the intracellular availability of cobalamin such as the serum concentration of HCY and MMA should ideally be included when assessing cobalamin status.^31^ Cobalamin is stable in serum even if samples are not strictly protected from light.^32^ Thus, in clinical practice, measurement of serum cobalamin can be conveniently performed in serum samples that are archived for up to 5 days. However, the long‐term (>5 days) stability of cobalamin in serum samples has not been reported.

#### 3.1.2 Serum HCY concentration

Serum HCY concentrations can be measured by gas chromatography‐mass spectrometry (GC/MS), with RIs of 5.0‐22.1 μmol/L^27^ and 5.9‐31.9 μmol/L.^33^ In human medicine, other methods that are used for measuring HCY are a chemiluminescent microparticle immunoassay and an enzyme‐cycling assay (Homocysteine Cobas C, Integra, Roche).

Homocysteine is a sulfurated intermediate amino acid that is produced from dietary methionine and is then either remethylated to methionine or metabolized to cysteine.^34^, ^35^, ^36^ Cobalamin (vitamin B_12_), folic acid (vitamin B_9_), and pyridoxine (vitamin B_6_) are essential cofactors for enzymes involved in the metabolism of HCY.^37^ Hyperhomocysteinemia thus reflects an insufficient intracellular availability of cobalamin, folic acid, or both for the synthesis of methionine, and HCY is a sensitive marker for the detection of intracellular B vitamin deficiency.^37^, ^38^ Serum HCY concentrations increase early in the course of cobalamin deficiency, and hyperhomocysteinemia can contribute to the development of clinical signs caused by the decreased availability of cobalamin for cellular metabolism.^6^ However, increased serum or plasma HCY concentrations are less specific for intracellular lack of cobalamin than is increased serum MMA concentrations.^39^ Serum HCY concentrations can also be increased with renal insufficiency^40^ or hypothyroidism.^41^

In humans, HCY has direct toxic effects on neurons and endothelial cells, can induce DNA strand damage, oxidative stress, and apoptosis^36^, ^42^ and also induce hepatic degeneration and fibrosis.^43^ Even small changes in serum HCY concentrations (≥5‐10 μmol/L) increase the risk of cardiovascular diseases in people.^36^, ^37^ Increased serum HCY concentrations occur in dogs with cardiac or renal disease.^44^

Circulating HCY is mostly bound to albumin. As a consequence, diseases associated with hypoalbuminemia, such as protein‐losing enteropathy (PLE), might be associated with a lower degree of hyperhomocysteinemia than expected or even with normohomocysteinemia.^38^, ^45^ However, it is likely that breed‐specific effects exist. For example in Greyhounds,^38^ and breed‐specific RIs for serum HCY concentrations might need to be established because other breeds of dog could also fail to show an association between hypocobalaminemia and hyperhomocysteinemia. Preanalytical factors are also important because serum HCY concentrations can be falsely increased if the separation of red blood cells from serum is delayed.^46^

#### 3.1.3 Serum MMA concentration

Methylmalonic acid concentrations can be determined in serum and urine samples. Serum MMA concentrations are measured routinely in human medicine by either a GC/MS or liquid chromatography‐tandem mass spectrometry (LC/MS‐MS) assay. However, measurement of serum MMA concentrations is currently not routinely performed in companion animals. RIs for serum MMA concentrations in dogs are 415‐1,193 nmol/L (Texas A&M University Gastrointestinal Laboratory [http://www.vetmed.tamu.edu/gilab]) and 393‐1,476 nmol/L.^47^ Serum samples are needed for measuring cobalamin; thus, specimens collected and used for serum chemistry analysis can also be used for measurement of both cobalamin and MMA.

Because MMA production increases as a result of decreased intracellular availability of cobalamin, MMA is a useful marker of cobalamin deficiency at the cellular level. Such a deficiency can occur due to cobalamin malabsorption, deficient cobalamin transport (which is not reported in dogs), depletion of cobalamin stores in the liver and kidneys secondary to cobalamin malabsorption, or a combination of these, leading to insufficient amounts of cobalamin entering the cells. Tissues or cells with a high turnover rate such as enterocytes or blood cells are primarily affected. Dogs with a serum cobalamin concentration below RI have significantly higher serum MMA concentrations compared to dogs with normocobalaminemia, but serum MMA concentrations are increased in some (12%) normocobalaminemic dogs.^26^ In cobalamin‐deficient Shar‐Peis, where a suspected hereditary but late‐onset form of cobalamin deficiency with mostly signs of GI disease exists, serum MMA concentrations can even be 10 times higher than in cobalamin‐deficient dogs of other breeds.^27^

Careful interpretation of serum MMA concentrations should be exercised in the presence of other conditions that can affect serum MMA concentrations, such as renal insufficiency causing increased serum MMA concentrations.^48^ Therefore, serum concentrations of creatinine, symmetric dimethylarginine, or both should also be evaluated when interpreting serum MMA concentrations.^49^ Other possible causes for increased serum MMA concentrations, unrelated to primary cobalamin deficiency and renal disease, are dehydration, small intestinal (SI) dysbiosis,^26^, ^50^ and inherited defects of the enzyme systems involved in the metabolism of cobalamin and MMA. Inherited etiologies also include defects in the absorption and transport of cobalamin such as a deficiency of transcobalamin II. Such defects have been reported in people, but further studies are needed to investigate whether dogs are also affected by these.

Of healthy normocobalaminemic Border Collies, 38% have increased urine MMA concentrations.^6^ None of these dogs have increased serum HCY or decreased cobalamin concentrations compared to cobalamin‐deficient dogs. These cobalamin‐deficient Border Collies have a primary methylmalonic aciduria. However, bacterial contamination of urine also has to be considered as a cause for increased urinary MMA concentrations.^47^ A similar presentation occurs in children with a defect in the methylmalonyl‐CoA mutase.^37^, ^51^, ^52^

Urine MMA concentrations are also measured by GC/MS^26^ or LC/MS‐MS.^47^ The RI was determined as 0‐4.2 mmol/mol of creatinine in 1 report.^6^ Dogs normally excrete <10 mg MMA/g of creatinine (<9.6 mmol MMA/mol of creatinine).^1^ Normalizing urine MMA to creatinine concentration minimizes the concentration or dilution effect of urine on MMA concentrations. An advantage of measuring urinary MMA concentration is easier determination because MMA concentrations in urine are up to 40‐fold higher than in serum. Furthermore, MMA is stable in urine, and sample collection is less invasive compared to the collection of blood samples.^6^

### 3.2 Interpretation of cobalamin status

The currently recommended interpretation of the cobalamin status in dogs is summarized in Table 2 (the cutoff serum cobalamin concentrations proposed below are based on the assay offered through the Gastrointestinal laboratory at Texas A&M University) and Fig. 4.

**Table 2:** Evaluation of the cobalamin status in dogs

	Cobalamin status			
	Normocobalaminemia	Hypocobalaminemia	Cobalamin deficiency	Hypercobalaminemia
Serum cobalamin concentration	Within RI	↓ Below RI	Undetectable	↑ Above RI
Serum HCY concentration	Within RI	Within RI or ↑ Above RI	Within RI or ↑ above RI	Not reported
Serum MMA concentration	Within RI#	Within RI or ↑ Above RI	↑ Above RI	Not reported

Note: ↓ indicates decreased; ↑ indicates increased.

Abbreviations: HCY, homocysteine; MMA, methylmalonic acid; RI, reference interval.

# Serum MMA concentration above RI might indicate insufficient intracellular (subclinical) cobalamin supply.

**Figure 4:**
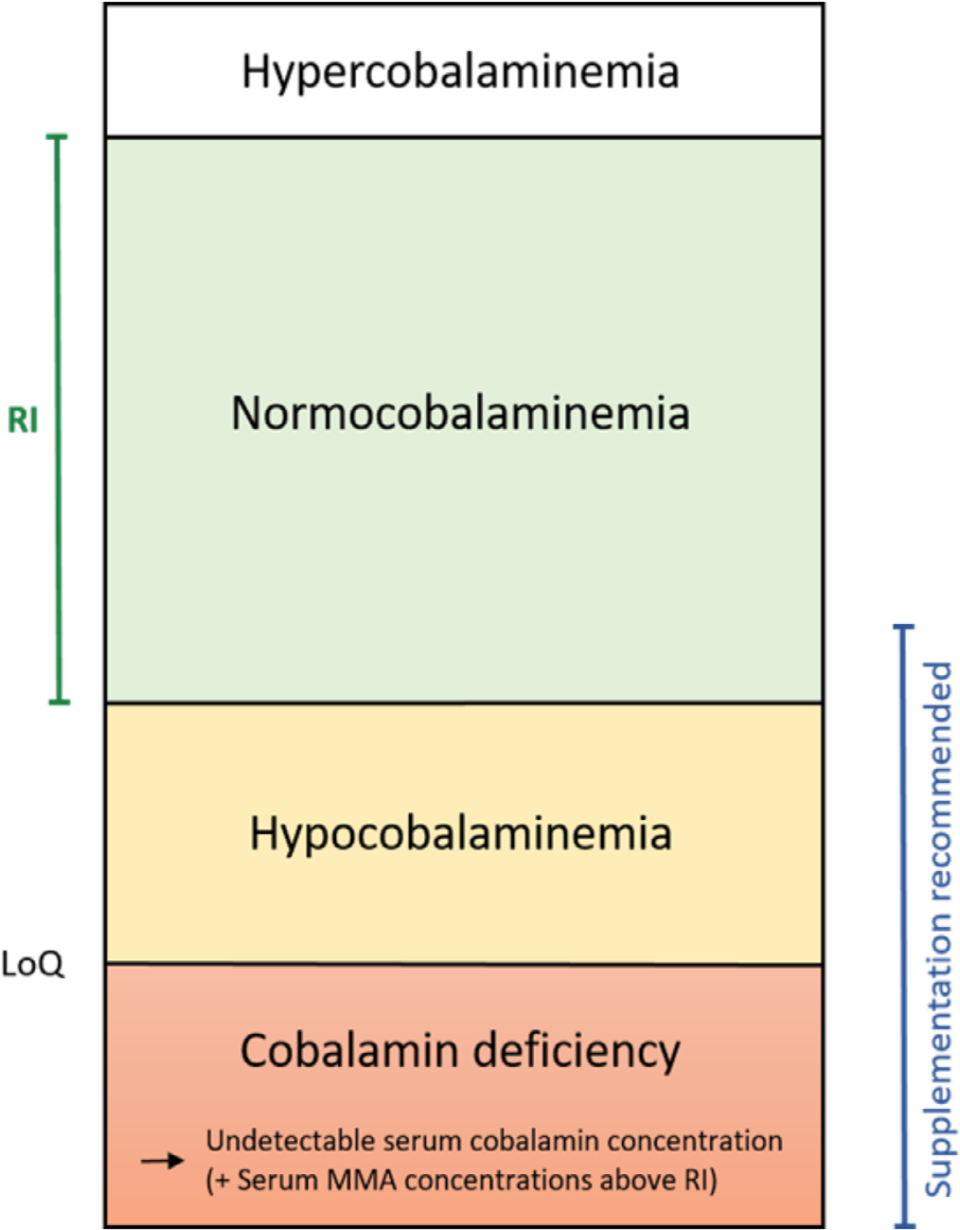
Interpretation of the cobalamin status in dogs. Hypocobalaminemia is typically referred to as a serum cobalamin concentration between the lower limit of quantification (LoQ) of the assay and the lower reference limit. Dogs with cobalamin deficiency have an undetectable serum cobalamin concentration (ie, below LoQ) and a serum MMA concentration above RI. Cobalamin should be supplemented whenever serum cobalamin concentration is suboptimal (ie, less than approximately 400 ng/L)

#### 3.2.1 Normocobalaminemia

Normocobalaminemia refers to a serum cobalamin concentration within the RI for healthy dogs. However, serum cobalamin concentration does not accurately reflect the cobalamin status on the cellular level. If cobalamin transport into the cell is hindered, intracellular cobalamin deficiency might result while the cobalamin concentration in serum remains within normal limits.^53^ In human medicine, standard assessment of cobalamin status includes measurement of serum cobalamin, HCY, MMA, and holotranscobalamin.^54^ Holotranscobalamin is the biologically active fraction of cobalamin that is bound to transcobalamin II. A canine holotranscobalamin assay is currently not available; therefore, measurement of serum MMA and HCY in addition to serum cobalamin is recommended (but currently not widely available) to assess cobalamin status in dogs.

Twelve percent of dogs with chronic enteropathy and a serum cobalamin concentration within the normal RI have an increased serum MMA concentration.^26^ Twenty‐two percent of dogs with cobalamin close to the lower end of the RI have increased serum MMA concentrations.^33^ These data suggest that a subtle or mild subclinical intracellular cobalamin deficiency exists in these dogs. In humans with suboptimal cobalamin status, the response to cobalamin supplementation is a marked reduction in serum MMA concentration.^55^ Dogs with serum cobalamin concentrations at the lower end of the RI have a normalization of serum MMA concentrations after cobalamin supplementation.^33^ This could indicate that the discrepancy between an increased serum MMA and a normal cobalamin concentration reflects intracellular cobalamin deficiency despite that body stores of cobalamin are sufficient to maintain normocobalaminemia. Such a “cobalamin resistance” occurs in geriatric, diabetic, and hemodialysis human patients.^53^ Alternative explanations could be the shift to an increased pool of circulating cobalamin with hepatic disease^53^ or a compromised translocation of cobalamin to the intracellular space (which is not reported in dogs).

#### 3.2.2 Hypocobalaminemia

Hypocobalaminemia is typically referred to in dogs with a serum cobalamin concentration between the lower limit of quantification (LoQ) of the assay and the lower reference limit (Fig. 4). However, careful interpretation is necessary because different laboratories use different LoQs (Table 1).

Thirty‐one percent of hypocobalaminemic dogs (as defined above) have serum MMA concentrations above the RI,^26^ and these dogs are assumed to be cobalamin‐deficient on the cellular level. However, it is possible that other factors (such as acute secondary SI dysbiosis due to an acute flare of the underlying enteropathy, proton‐pump inhibitor use,^56^ or administration of oral antibiotics—Manchester AC, Blake A, Webb C, Lidury J, Steiner J, Suchodolski J. Tylosin causes dysbiosis associated with altered fecal unconjugated bile acids in healthy dogs. Proceedings of the ACVIM Forum 2019, GI11 [abstract])^57^ contributed to the suboptimal serum cobalamin, but normal serum MMA concentrations in the remaining 69% of dogs and that the hypocobalaminemia in these dogs did not reflect a true whole‐body cobalamin deficiency. However, it remains to be determined whether the hypocobalaminemia associated with SI dysbiosis correlates with cobalamin deficiency at the cellular level.

After supplementation, cobalamin is rapidly extracted from plasma by tissues where it binds to cobalamin‐dependent enzymes with a relatively long half‐life.^58^ Thus, it is possible that the half‐life of cobalamin that is bound intracellularly as a coenzyme exceeds the residence time of cobalamin within the circulation, which would mean that cobalamin continues to be active in the tissues and cobalamin‐dependent metabolic processes are able to occur despite low serum cobalamin concentrations. This is often observed in the treatment of dogs with inherited disorders of cobalamin metabolism.^59^

#### 3.2.3 Cobalamin deficiency

The term cobalamin deficiency is not well‐defined in the veterinary literature. Most current cobalamin assays have an LoQ between 45 and 150 ng/L (Table 1). We propose using the term cobalamin deficiency for dogs with an undetectable serum cobalamin concentration and a serum MMA concentration above RI (Table 2, Figure 4).

Sixty‐three percent of dogs with undetectable serum cobalamin concentrations have increased serum MMA concentrations indicating cellular cobalamin deficiency.^26^ Normal serum MMA concentrations in the remaining 37% of dogs with an undetectable serum cobalamin concentration might suggest that a sufficient amount of cobalamin as a cofactor is bound to cobalamin‐dependent enzymes and a normal activity of these enzyme is maintained in these dogs, whereas cobalamin concentrations in the circulation are already severely decreased because of their much shorter half‐life.^58^ Therefore, measurement of serum cobalamin concentration alone is not sufficient to diagnose or exclude intracellular cobalamin deficiency, and determination of serum MMA (and HCY) is recommended in addition to serum cobalamin concentration.

#### 3.2.4 Hypercobalaminemia

Hypercobalaminemia refers to a serum cobalamin concentration above the normal RI. Until recently, hypercobalaminemia was considered as a benign finding and was basically ignored in companion animals. However, recent data in people suggest that hypercobalaminemia is an underestimated finding that can reflect a number of serious underlying diseases such as solid neoplasms, hematological and other malignancies, and hepatic or renal diseases.^60^, ^61^, ^62^ Abnormally high serum cobalamin concentrations occur in cats with hepatic and neoplastic diseases.^63^ A retrospective analysis of the medical records and data from 592 dogs that were presented for further diagnostic workup at the University of Leipzig Small Animal Clinic showed that 2% (n = 11) of all dogs in which serum cobalamin status was determined and that did not receive any supplemental cobalamin (n=487 dogs) had a serum cobalamin concentration above RI, ranging from 1,100‐3,561 ng/L (median: 1,334 ng/L) (unpublished data). These hypercobalaminemic dogs had mostly (73%) signs of chronic GI disease, and a neoplastic condition could not be definitively excluded in 82% of the dogs. These findings suggest that hypercobalaminemia is less frequently observed in dogs than in cats,^63^ in which a prevalence of 14% (28 of 202 cats) was observed in the study above (unpublished data), and that hypercobalaminemia might be of clinical relevance in dogs that have not received supplemental cobalamin. A higher prevalence (36%) of hypercobalaminemia is reported (Bertolani C, Tabar MD, Esparza A, et al. Hypercobalaminemia in 144 samples from cats and dogs. *J Vet Intern Med*. 2019;33(2):1070‐1071 [abstract]) in a smaller number of dogs with clinical signs similar to those reported above (unpublished data), the upper reference limit in which was also much lower (590 ng/L) which might at least in part explain the discrepancy between the reported prevalence of hypercobalaminemia in dogs. Measurement of serum cobalamin concentrations at 2 different laboratories also needs to be considered when comparing the prevalence of hypercobalaminemia between these 2 studies. Serum MMA concentrations have not been evaluated in dogs with hypercobalaminemia.

## 4 CONDITIONS ASSOCIATED WITH A SUBOPTIMAL COBALAMIN STATUS

Hypocobalaminemia and cobalamin deficiency can have a variety of causes in dogs including exocrine pancreatic insufficiency (EPI), chronic severe ileal disease (eg, infiltrative disorders such as idiopathic inflammatory bowel disease [IBD], alimentary lymphoma, or GI histoplasmosis), SI dysbiosis (though this condition is rarely definitively diagnosed as the acquisition and analysis of the SI microbiota is cumbersome and invasive), and inherited conditions (Fig. 1B).^49^, ^64^, ^65^, ^66^, ^67^, ^68^, ^69^, ^70^, ^71^ Hypocobalaminemia also occurs in people with IBD (Crohn's disease and ulcerative colitis). There are no reported cases of cobalamin deficiency of dietary origin in dogs.^31^

### 4.1 Clinicopathological findings and clinical presentation

Blood cell count abnormalities, typically nonregenerative anemia with megaloblastosis, neutropenia, and hypersegmented neutrophils, can be early signs of derangements in cobalamin metabolism.^72^ With progression, disorders of phospholipid and amino acid metabolism have been documented in other species and might also occur in dogs.^21^, ^73^

In addition to blood cell dyscrasias and organic acidemias associated with neurological signs, especially dogs with hereditary cobalamin deficiency can show severe clinical signs such as failure to thrive, anorexia, lethargy, vomiting, and diarrhea. The age of affected dogs at presentation with clinically apparent disease also varies, with the majority of dogs showing clinical signs early in life and a smaller proportion of dogs having a more delayed onset of clinical signs.

### 4.2 Hereditary conditions

A genetic background for cobalamin deficiency has been confirmed or suspected in Giant Schnauzers,^1^, ^74^ Border Collies,^18^, ^75^, ^76^, ^77^, ^78^ Chinese Shar‐Peis,^79^, ^80^ Beagle dogs,^17^, ^59^, ^81^, ^82^, ^83^ Australian Shepherd dogs,^19^, ^25^ a Yorkshire Terrier,^20^ and also a mixed breed dog (Maguire D, Solano‐Gallego L, English K, et al. Cobalamin deficiency in a collie cross Bedlington Terrier. Available at: https://www.esvcp.org/index.php/docman/clin-path-cases/106-case-2-maguire-with-diagnosis/file.html); genetic defects and hypocobalaminemia has been confirmed for a few canine breeds. Certain dog breeds have a predisposition for hypocobalaminemia for which the underlying mechanism or cause has not been elucidated (see Section 4.2.1.). In some of these breeds, cobalamin deficiency is a genetic trait. This condition is classified as a hereditary or selective disorder in cobalamin metabolism (see Section 4.2.2.). Finally, a genetic cause (eg, a specific mutation of the cubam receptor) of selective cobalamin malabsorption was identified or confirmed in some affected dog breeds (see Section 4.2.3). The current literature is reviewed here, but it should be noted that additional dog breeds might have a predisposition for cobalamin deficiency because current studies do not cover an exhaustive list of geographical areas.

#### 4.2.1 Breed predispositions for hypocobalaminemia and cobalamin deficiency

Serum concentrations of cobalamin have been used for many years in veterinary practice to identify dogs with hypocobalaminemia or cobalamin deficiency, and 2 large surveys have been conducted with the aim to identify canine breeds that might have a predisposition to develop cobalamin deficiency.^70^, ^84^

Test results of serum samples from 9,960 dogs submitted over a 13‐year period to a laboratory in the United Kingdom were analyzed to determine breed predispositions for hypocobalaminemia, hypofolatemia, or both.^84^ Breeds of dogs with an increased frequency of hypocobalaminemia were the Chinese Shar‐Pei, Staffordshire Bull Terrier, German Shepherd Dog, and mixed breed dogs. Golden Retrievers had a decreased odds ratio for being diagnosed with hypocobalaminemia. There is an increased frequency of hypocobalaminemia in Chinese Shar‐Peis and German Shepherd Dogs in North America.^70^ A high prevalence of severe hypocobalaminemia in Stafford Bull Terriers and mixed breed dogs is reported in the United Kingdom,^84^ but Staffordshire Bull Terriers do not have an increased risk of hypocobalaminemia in the United States.^70^

In the North American study,^70^ serum cobalamin test results from 28,675 dogs of 164 breeds were reviewed to identify breeds with an increased risk of being identified with hypocobalaminemia. This study found Greyhounds to have the highest proportion of serum samples submitted for cobalamin analysis and also the lowest serum cobalamin concentrations compared to the other 18 breeds of dogs that were significantly more likely to have a subnormal serum cobalamin concentration. This suggests that hypocobalaminemia or cobalamin deficiency occurs frequently in this breed, which is supported by another study indicating that Greyhounds frequently have hypofolatemia with or without hypocobalaminemia.^38^ The high percentage of affected dogs with hyperhomocysteinemia in this study suggests that cobalamin deficiency is common in this breed.^38^ However, it is also possible that normal serum cobalamin concentrations in Greyhounds are lower than in dogs of other breeds, thus calling for a breed‐specific RI. An effect of exercise is unlikely because Greyhounds included in the study were all retired racing dogs.^38^

#### 4.2.2 Selective cobalamin malabsorption

Selective cobalamin malabsorption is a rare congenital disease. The first report about selective cobalamin malabsorption in dogs describes 2 related Giant Schnauzers that lacked the expression of the cobalamin‐IF‐receptor complex in the apical brush border of the ileal and renal epithelium.^1^ The human analog of this rare genetic disorder is called Imerslund‐Gräsbeck syndrome (IGS). Dogs with IGS are unable to absorb dietary cobalamin.^1^, ^17^, ^74^

Classical clinical signs of IGS such as intermittent diarrhea, inappetence, poor body condition, and failure to grow typically manifest within the first year of age. Around that time fetal stores of cobalamin are depleted while growth demands cobalamin to be utilized at high rates. Dogs might be misdiagnosed because of the secondary effects of hypocobalaminemia on metabolism such as the development of hypoglycemia, ketoacidosis, and hyperammonemia.^18^, ^19^, ^78^ Therefore, cobalamin deficiency should be considered as a differential diagnosis in dogs presented with a suspicion of a portosystemic shunt, hydrocephalus, or metabolic disorders such as hypoglycemia and renal insufficiency.^17^

Another possible clinical manifestation of IGS is intermittent impaired swallowing.^76^ Mucosal ulcerations might also develop,^76^, ^85^ but glossitis, glossodynia, and stomatodynia are reported only in people with cobalamin deficiency. Additionally, bradyarrhythmia is a possible clinical sign in dogs.^76^ Skin lesions (reversible hyperpigmentation) occur in children with IGS.^86^

Hematologic abnormalities in human patients with IGS include a dyshematopoesis that is typically characterized by a nonregenerative anemia of varying severity and neutropenia. Microscopically, hypersegmented neutrophils may be noted. Megaloblastosis is rarely seen in dogs,^75^ and some dogs with IGS present with a normal hemogram.^76^ Although children with this disorder develop macrocytic anemia, the anemia in dogs is usually normocytic and normochromic.^72^, ^87^, ^88^, ^89^ Thus, megaloblastic anemia does not appear to be as closely associated with cobalamin deficiency in dogs as it is in people. In affected dogs, both large and small red blood cells are present systemically, resulting in an increased red cell distribution width while the mean cellular volume remains within RI.^72^ Rarely, dogs with IGS can present with Heinz body anemia caused by hyperhomocysteinemia.^78^

Breed‐specific changes in serum cobalamin concentrations and distinct clinical manifestations exist in the Border Collie, Beagle, and Chinese Shar‐Pei breeds (Table S1).

##### Border Collie

Several case reports and case series have described a selective cobalamin malabsorption in the Border Collie breed.^18^, ^75^, ^76^, ^78^ Clinical manifestations in some cases are mild and the onset of clinical signs can be delayed into early adulthood compared to other breeds with selective cobalamin malabsorption.^17^, ^75^ Also, juvenile cobalamin deficiency might not be routinely associated with hematologic changes in the Border Collie breed.^18^, ^75^, ^76^

About 40% of healthy Border Collies have increased urine MMA levels,^6^ but none of the normocobalaminemic dogs in that study had increased plasma HCY concentrations compared to the Border Collies with cobalamin deficiency. It has been hypothesized that these dogs have a primary methylmalonic aciduria.^6^ Of note, hypocobalaminemia is also associated with an increased prevalence of EPI in Border Collies.^70^

##### Beagle

Hereditary cobalamin malabsorption is reported in Beagle dogs,^17^, ^59^, ^85^, ^90^ and 2 Beagles with selective cobalamin malabsorption developed degenerative liver disease.^83^ The hepatopathies seen in these dogs might be associated with the chronic hyperhomocysteinemia which was demonstrated to promote hepatic inflammation and fibrosis in an experimental study in rats.^43^ Degenerative hepatopathies are also well described in farm animals, especially in lambs and goats with decreased cobalt intake causing hypocobalaminemia, but this etiology has not been reported in dogs with hereditary cobalamin malabsorption.^83^

Decreased phagocytic activity has been demonstrated in human patients with cobalamin deficiency.^91^ Cobalamin‐deficient Beagle dogs are suspected to be immunosuppressed, supporting the development of complicating infections such as fungal hepatopathy or necrotizing pneumonia.^78^, ^81^, ^92^

##### Chinese Shar‐Pei

Chinese Shar‐Peis have a high prevalence of cobalamin deficiency compared to other canine breeds.^27^, ^79^, ^80^, ^93^ Even apparently healthy Chinese Shar‐Peis have increased serum MMA concentrations compared to healthy dogs of other breeds, which led to the hypothesis that some healthy Chinese Shar‐Peis have subclinical cobalamin deficiency.^80^ Cobalamin deficiency in Chinese Shar‐Peis is also associated with hyperhomocysteinemia.^27^ Clinical signs of affected dogs reflect primarily an involvement of the GI tract with chronic signs of GI disease such as diarrhea, vomiting, and weight loss.^93^ Classical signs of cobalamin deficiency such as hyperammonemia, hypoglycemia, and seizures are typically lacking in these dogs.^79^

Hypocobalaminemia in Chinese Shar‐Peis is not associated with other common diseases in this breed such as cutaneous mucinosis or Shar‐Pei fever.^93^ However, evaluation of several biomarkers of inflammation suggests the existence of an inflammatory phenotype in both hypocobalaminemic and normocobalaminemic Chinese Shar‐Peis.^93^ This might reflect compromised GI health in affected dogs, with the result of a low‐grade inflammation and malabsorption of vitamins such as cobalamin.

##### Komondor

A recent study reported that selective cobalamin malabsorption is also inherited via an autosomal recessive trait in Komondor dogs, an old Hungarian herding dog breed.^94^

#### 4.2.3 Genetic causes of selective cobalamin malabsorption

Each of the 2 subunits of the cubam receptor complex (ie, AMN and CUBN) depends on the normal structure of the other part of the receptor for epithelial brush border expression and function.^81^ Several (close to 400) *AMN* and *CUBN* mutations occur in people with IGS. In dogs, only 2 *AMN* mutations and 4 *CUBN* mutations have been identified to date.

Cubam expression is disrupted by 2 independent mutations in the *AMN* gene in Australian Shepherd dogs and Giant Schnauzers.^25^ A *CUBN* mutation causes IGS in Border Collies.^77^, ^95^ A *CUBN* mutation is also responsible for canine IGS in the Beagle breed.^82^ The *CUBN* mutation in juvenile Beagle dogs causes more severe cobalamin malabsorption than a different *CUBN* defect in Border Collies^81^ and the former condition to resemble more closely IGS caused by *AMN* mutations in Giant Schnauzers and Australian Shepherd dogs. A residual CUBN expression, which does not exist in affected Beagle dogs, might explain why the diagnosis in some Border Collies can be delayed compared to affected dogs of other breeds.^81^ Furthermore, in a study including 200 randomly selected Border Collies, the frequency of dogs being heterozygous for the *CUBN* mutation was relatively low at 6.2%,^77^ whereas increased carrier frequency has been noted in Beagles, but the disease occurrence might be geographically dependent.^82^, ^90^ In Komondor dogs, a *CUBN* splice‐site variant causes cobalamin deficiency.^94^ Genetic tests have become commercially available and allow for a diagnosis prior to the clinical manifestation of the condition and also the detection of asymptomatic carriers to guide breeding decisions (https://centerofanimalgenetics.com, https://animalabs.com, or http://www.laboklin.de). A large genomic study in Chinese Shar‐Peis revealed an association of 2 canine microsatellite markers, located on canine chromosome 13, with cobalamin deficiency in this breed.^79^ However, there are no previously identified genes in this chromosomal region that are associated with cobalamin deficiency in dogs or other species.^79^

### 4.3 Gastrointestinal diseases

#### 4.3.1 Exocrine pancreatic insufficiency

Cobalamin deficiency is common in dogs with EPI. A study showed 82% of dogs with EPI have decreased serum cobalamin concentrations, and 36% of these dogs had severe hypocobalaminemia (defined as a serum cobalamin concentration of <100 ng/L in this study).^66^ A breed predisposition for EPI exists in German Shepherd Dogs, Rough Coated Collies, Chow‐Chows, Cavalier King Charles Spaniels, and West Highland White Terriers, with German Shepherd Dogs representing about 60% of all cases of EPI.^66^ Pancreatic acinar atrophy is also an inherited disorder in the Eurasian breed.^96^

Exocrine pancreatic insufficiency is characterized by an inadequate production of digestive enzymes from pancreatic acinar cells, leading to the typical clinical signs of weight loss in the face of polyphagia and increased fecal volume.^97^, ^98^ Failure to absorb cobalamin in dogs with EPI might be caused by 4 postulated or hypothetical mechanisms: pancreatic secretion of IF is reduced or absent, digestive enzymes are lacking causing an impaired release of cobalamin from haptocorrin and thus no binding of cobalamin to IF, secondary SI dysbiosis compromising the endogenous production of cobalamin, and the intestinal mucosa might also be compromised by the presence of toxic metabolites due to SI dysbiosis.^9^, ^99^, ^100^, ^101^ However, in people cobalamin assimilation does not improve with antibiotic treatment,^102^ and there is no data that demonstrate a benefit of probiotics (eg, *Enterococcus faecium* SF68^103^) or prebiotics.

Because the exocrine pancreas is the major site of IF synthesis in dogs, cobalamin supplementation is often necessary in these dogs. However, cobalamin deficiency in some cases is not corrected by enzyme replacement alone likely because IF is not consistently a component of the exogenous enzyme mixture.^99^ Treatment with bovine pancreatic enzyme extract is also not sufficient to restore cobalamin absorption in dogs with EPI, because IF appears to be species‐specific. The fact that serum cobalamin concentrations <100 ng/L are a negative prognostic factor^66^ underlines the importance to adequately supplement dogs with EPI, but only 4 of 135 (3%) hypocobalaminemic dogs with EPI were given supplemental cobalamin in that study.^66^ Another recent study revealed a prevalence of hypocobalaminemia (serum cobalamin concentration <350 ng/L) of 55% in dogs with EPI.^104^ Hypocobalaminemia was also shown to be a negative prognostic factor in that study, and 112 of 116 dogs (82%) received cobalamin supplementation.^104^

#### 4.3.2 Chronic inflammatory enteropathies

Chronic inflammatory enteropathies (CIE), further classified based on the response to treatment as either food‐responsive enteropathy, antibiotic‐responsive enteropathy, immunosuppressant‐responsive enteropathy (IRE), or nonresponsive enteropathy (NRE), are characterized by chronic persistent or recurrent clinical signs of GI disease (such as vomiting, diarrhea, weight loss, or a combination of those) for longer than 3 weeks and are diagnosed retrospectively based on the response to treatment.^65^, ^71^

It has been postulated that chronic mucosal disease affecting the ileum reduces the epithelial expression or function of the cubam receptor leading to a reduced mucosal uptake of cobalamin. However, currently there are no studies that have investigated the expression levels of the ileal cobalamin‐IF‐receptor in dogs with chronic enteropathies.

In addition, cobalamin has been hypothesized to be decreased in dogs with CIE because of secondary SI dysbiosis. Once the body stores of cobalamin are depleted, cobalamin deficiency can occur.^105^ The half‐life of cobalamin in healthy dogs is approximately 6‐16 weeks.^106^ It is assumed that this half‐life is decreased in dogs with CIE as a result of a reduced cobalamin absorption in the face of normal biliary excretion and enterohepatic circulation.^49^

Reported prevalences of hypocobalaminemia in dogs with CIE ranged from 19% to 38%,^49^, ^65^, ^107^, ^108^, ^109^, ^110^ and similar findings have been reported in cats.^111^ Dogs with suboptimal serum cobalamin status might not respond to treatment of the primary disease process unless given supplemental cobalamin, because cobalamin is essential for many cell functions and mucosal regeneration, and a deficiency can contribute to mucosal inflammatory infiltration and villous atrophy.^72^, ^105^

##### Correlation of hypocobalaminemia with histopathologic findings

Histological lesions in dogs with CIE are often multifocally distributed, and different degrees of disease severity might be found among the different segments of the intestinal tract and also among different regions within the same segment (Dossin O, Tesseydre JF, Concordet D. Is duodenal mucosa representative of other small intestinal parts in inflammatory bowel disease affected dogs? J Vet Intern Med. 2007;21:613 [abstract]).^112^, ^113^, ^114^ Because histological lesions of CIE might be found in the ileum in the face of a normal duodenal mucosa, ileal biopsies should be routinely collected in addition to duodenal biopsies in all dogs with chronic signs of GI disease undergoing GI endoscopy.^113^, ^114^

Hypocobalaminemia correlates with an increased number of intraepithelial lymphocytes in the ileal mucosa,^114^ presenting evidence of an association between ileal mucosal inflammatory changes and hypocobalaminemia in dogs with CIE. However, the expression of the cobalamin‐receptor in dogs with CIE has not been evaluated.

##### Correlation of hypocobalaminemia with outcome in dogs

Several factors have been evaluated for their potential to predict outcome in dogs with CIE.^65^ In addition to the presence of severe mucosal lesions in the duodenum and hypoalbuminemia (serum albumin concentration of <20 g/L), hypocobalaminemia (defined as a serum cobalamin concentration of <200 ng/L in this study) at the time of diagnosis might predict refractoriness to treatment and was strongly correlated with a negative outcome despite supplementation of cobalamin for 6 weeks.^65^ However, despite that hypocobalaminemia is a negative prognostic factor, adequate supplementation of cobalamin appears to be essential for the success of treatment. Thus, further studies need to investigate if early initiation of cobalamin supplementation is associated with a better outcome in dogs with CIE. Hypocobalaminemia also correlates with hypoalbuminemia in dogs with CIE.^65^, ^108^, ^109^

##### Protein‐losing enteropathy

Protein‐losing enteropathy is a syndrome associated with an abnormal loss of albumin into the intestinal lumen. Common causes for PLE are altered lymphatic drainage or increased mucosal permeability.^69^, ^115^ A predisposition for primary intestinal lymphangiectasia exists in Rottweilers, Yorkshire Terriers, Chinese Shar‐Peis, Maltese, Norwegian Lundehunds, Basenjis, Soft Coated Wheaten Terriers, and German Shepherd Dogs.^115^, ^116^, ^117^, ^118^, ^119^, ^120^, ^121^, ^122^, ^123^ Secondary intestinal lymphangiectasia is associated with mucosal inflammation in IRE/NRE, intestinal neoplasia, or infectious diseases (eg, GI histoplasmosis) in dogs.^69^ Prevalences of hypocobalaminemia range from 43%‐75% in dogs with non‐neoplastic, noninfectious causes of PLE.^65^, ^107^, ^124^ Hypocobalaminemia was also associated with decreased serum alpha_1_‐proteinase inhibitor concentrations (presumably due to PLE) in Yorkshire Terriers.^125^

Serum cobalamin concentrations should be evaluated in all dogs with PLE and, if needed, supplemental cobalamin be started early.^69^

#### 4.3.3 Alimentary lymphoma

Sixteen percent of dogs with multicentric lymphoma have hypocobalaminemia, and this finding is associated with a poor outcome.^68^ A higher rate of hypocobalaminemia (40% and 71%) is seen in dogs with low‐grade (lymphocytic) GI lymphoma.^126^, ^127^ It is presumed that the hypocobalaminemia in dogs with lymphoma is a consequence of the ileal infiltration with neoplastic lymphocytes hypothetically resulting in a disruption of the receptor‐mediated GI uptake of cobalamin.^68^

#### 4.3.4 Small intestinal dysbiosis

Small intestinal (SI) dysbiosis is defined as an altered composition, richness, or both of the intestinal microbiota.^50^

Competition between bacteria and host cells for essential nutrients such as cobalamin can lead to malnutrition.^67^ *Bacteroides* spp. are the principle competitors for cobalamin with the host because of the ability to utilize cobalamin‐IF complexes, in contrast to other bacteria that can only bind free cobalamin.^128^ If these folate‐producing bacteria localize to the proximal intestine, an increased amount of folate of bacterial origin can be absorbed by the host resulting in increased serum folate concentrations.^67^ Hence, hypocobalaminemia with concurrent hyperfolatemia might suggest the presence of SI dysbiosis. However, there are currently no studies using culture‐independent techniques that provide microbiological or ecological evidence of SI dysbiosis associated with systemic hypocobalaminemia or hyperfolatemia. The definitive diagnosis of SI dysbiosis remains challenging, and the diagnostic constellation of hypocobalaminemia with concurrent hyperfolatemia should not be used as a justification for antibiotic administration.

## 5 TREATMENT

Cobalamin should be supplemented whenever serum cobalamin concentration is subnormal. There is a 12% probability that CIE dogs with low‐normal serum cobalamin concentration (ie, less than approximately 400 ng/L) might also benefit from supplementation.^26^, ^33^ Cyanocobalamin has traditionally been used for supplementation, as it is widely available and inexpensive. The possibility of normocobalaminemia being restored in hypocobalaminemic dogs with CIE when treating the underlying disease without supplementing cobalamin has not been demonstrated.

In dogs with CIE and suboptimal cobalamin status, cobalamin is recommended to be given at a dose of 50 μg/kg SC every 7 days or 50 μg/kg PO q24h.^28^, ^33^ Current consensus is that oversupplementation with cobalamin or cobalamin toxicity does not occur. While this is a reasonable assumption based on theoretical considerations, there are no data available in companion animals to support this hypothesis. In people, chronic oversupplementation with cobalamin might increase the risk for lung cancer in smokers.^129^, ^130^ In dogs with IGS, dosing intervals are highly variable with cobalamin injections being done weekly, every other week, every 4 weeks, or bimonthly. In 1 case that was supplemented every 4‐5 months, clinical signs and methylmalonic aciduria returned.^1^ After starting cobalamin supplementation, appetite and well‐being of dogs with IGS can return to normal within 12 to 48 hours^74^, ^87^ and weight gain will follow over days to weeks. Normalization of hematological abnormalities can take up to 14 days.^74^, ^87^ Urinary MMA excretion as well as the serum MMA concentration can be expected to normalize (ie, decrease to within RI) within a week of initiating cobalamin supplementation.^17^, ^74^, ^87^ However, proteinuria in dogs with selective cobalamin malabsorption will persist lifelong due to abnormal renal tubular cubam function that is not corrected by cobalamin supplementation.^131^ In people with IGS, tubular proteinuria does not adversely affect renal function, and long‐term follow‐up of affected human patients (up to 50 years) suggests that renal prognosis is excellent.^132^

Dogs with selective cobalamin malabsorption—if left untreated—will die as a result of metabolic derangements and immunodeficiency.^78^, ^81^, ^83^, ^92^ There is limited evidence‐based information about the optimum route, dose, and dosing interval of cobalamin supplementation in dogs available. Currently either parenteral or oral cobalamin supplementation is recommended:

### 5.1 Parenteral cobalamin supplementation

#### 5.1.1 Protocol

Initially, weekly SC injections of 50 μg/kg SC cyanocobalamin (250‐1500 μg depending on the size of the dog; see Table S2) over a period of 6 weeks, then an additional dose 1 month later, followed by measurement of serum cobalamin concentration 1 month after the last dose.^33^

#### 5.1.2 Monitoring

If the underlying disease process has resolved and cobalamin body stores have been restored, serum cobalamin concentration should be above the RI at the time of retesting. If serum cobalamin concentration is still within RI (ie, supranormal serum cobalamin concentrations have not been achieved), cobalamin supplementation was traditionally recommended to be continued every 2‐4 weeks. More than 90% of dogs with a suboptimal serum cobalamin status achieve hypercobalaminemia within 4 weeks of parenteral cobalamin supplementation, but serum cobalamin concentrations decreased within the RI towards the 12‐week mark of cobalamin supplementation in the majority of these dogs.^28^ In some dogs, serum cobalamin concentrations are not adequate with cyanocobalamin supplementation, and hydroxocobalamin might be more effective in these dogs (Steiner JM, personal communication). However, monitoring serum MMA concentrations alone or in combination with serum cobalamin concentrations might be a more appropriate way to reevaluate the canine cobalamin status after treatment.^59^

A study investigating the effect of injectable hydroxocobalamin for the treatment of Beagle dogs with IGS showed a dose of 1 mg hydroxocobalamin administered IM to be adequate for maintaining a normal clinical status and normal urine MMA concentrations for up to 2 months.^59^ Hydroxocobalamin is considered the treatment of choice for children with IGS^88^, ^133^, ^134^ and other types of hereditary cobalamin deficiency.^135^, ^136^ It is likely more effective than cyanocobalamin because this compound presents the natural form of cobalamin.^137^ In addition, IM injections of hydroxocobalamin appear to cause less pain than cyanocobalamin.^134^

### 5.2 Oral cobalamin supplementation

#### 5.2.1 Protocol

Cobalamin (50 μg/kg PO) is administered once daily (250‐1000 μg in dogs, depending on the size of the dog; see Table S3) over a period of 12 weeks,^33^ followed by a recheck of serum cobalamin concentration after discontinuation of supplementation. Dogs were rechecked the day after the last PO cobalamin dose in this study,^33^ but the authors typically recheck serum cobalamin concentration 1 week after the last cobalamin tablet.

#### 5.2.2 Monitoring

Several studies in human patients with hypocobalaminemia suggest that supplementation with oral cobalamin might be as effective as parenteral administration.^138^, ^139^, ^140^ A study that included 51 dogs showed that serum cobalamin concentrations increased significantly and normocobalaminemia was achieved in all hypocobalaminemic dogs with chronic enteropathy after daily oral cobalamin supplementation for at least 3 weeks (20‐202 days).^141^ However, similar to this study, another investigation by the same research group showed that supranormal serum cobalamin concentrations were achieved in only about half of the dogs. The dogs in this study reached normal serum cobalamin concentrations within 4 weeks of receiving PO cobalamin, but a hypercobalaminemic state was reached after 12 weeks of PO cobalamin supplementation in about two thirds of these dogs.^28^ In addition, other recent studies suggest that daily PO cobalamin supplementation is effective to normalize the serum cobalamin status in dogs with CIE and also to maintain normal cobalamin status in dogs with hereditary cobalamin malabsorption that had previously been supplemented with parenteral hydroxocobalamin.^28^, ^47^ Thus, oral cobalamin supplementation is a simple, noninvasive, and pain‐free alternative to weekly SC injections of either cyanocobalamin or hydroxocobalamin.^28^, ^33^ An alternative pathway of intestinal cobalamin absorption beyond receptor‐mediated transport in the ileum is a possible explanation why oral cobalamin supplementation might be effective in spite of a defective receptor‐mediated uptake into enterocytes. This has also been reported in people.^11^ However, expression levels of the ileal cobalamin‐IF receptor in dogs with chronic enteropathies have not been investigated.

## CONFLICT OF INTEREST DECLARATION

Romy Heilmann has received speaker honoraria from Dechra company for presentations at continuing education meetings. Dechra has an oral cyanocobalamin formulation commercially available.

## OFF‐LABEL ANTIMICROBIAL DECLARATION

Authors declare no off‐label use of antimicrobials.

## INSTITUTIONAL ANIMAL CARE AND USE COMMITTEE (IACUC) OR OTHER APPROVAL DECLARATION

Authors declare no IACUC or other approval was needed.

## HUMAN ETHICS APPROVAL DECLARATION

Authors declare human ethics approval was not needed for this study.

## Supporting information


**Appendix** S1: Supporting information
