# Supplementary material for: Review of cobalamin status and disorders of cobalamin metabolism in dogs
Source: J Vet Intern Med. 2019 Nov 23;34(1):13–28. doi: 10.1111/jvim.15638 (PMC6979111; doi:10.1111/jvim.15638)
Supplement: Supplementary file 1 — Appendix S1: Supporting information [file JVIM-34-13-s001.pdf]

Supplementary Table 1: Variables determined in dogs of breeds with selective cobalamin malabsorption

	Hypocobalaminemia/ Cobalamin deficiency	Methylmalonic aciduria	Hyperhomocystein-emia
**Border Collie**			
Morgan et al.[75]	☑	☑	
Battersby et al.[18]	☑	☑	
Lutz et al.[76]	☑	☑	☑
Fyfe et al.[95]	☑	☑	
Owczarek-Lipska et al.[77]	☑	☑	☑
Fyfe et al.[81]	☑	☑	
**Beagle**			
Fordyce et al.[17]	☑	☑	
Kook et al.[83]	☑	☑	☑
Kook et al.[59]	☑	☑	
Kook et al.[47]	☑	☑	
**Chinese Shar-Pei**			
Grützner et al.[79]	☑		
Bishop et al.[80]	☑	☑	
Grützner et al.[27]	☑	☑	☑
Grützner et al.[93]	☑		
**Australian Shepherd dogs and Giant Schnauzers**			
He et al.[25]	☑	☑	
**Komondor dogs**			
Fyfe et al.[94]	☑	☑	

Supplementary Table 2: Parenteral supplementation of cobalamin in dogs

Body weight	<5 kg	5–10 kg	10–20 kg	20–30 kg	30–40 kg	40–50 kg	>50 kg
Recommended dose of cobalamin	250 µg	400 µg	600 µg	800 µg	1000 µg	1200 µg	1500 µg

(Reference: Texas A&M University Gastrointestinal Laboratory; www.vetmed.tamu.edu/gilab).

Supplementary Table 3: Oral supplementation of cobalamin in dogs

Weight	<10 kg	10–20 kg	>20 kg
Dose of Cobalamin	250 µg	500 µg	1000 µg

(Reference: Texas A&M University Gastrointestinal Laboratory; www.vetmed.tamu.edu/gilab).
